# Community-level trachoma ecological associations and the use of geospatial analysis methods: A systematic review

**DOI:** 10.1371/journal.pntd.0010272

**Published:** 2022-04-08

**Authors:** Clara R. Burgert-Brucker, Molly W. Adams, Pia Mingkwan, Rebecca Flueckiger, Jeremiah M. Ngondi, Anthony W. Solomon, Emma M. Harding-Esch

**Affiliations:** 1 RTI International, Washington, District of Columbia, United States of America; 2 London School of Hygiene & Tropical Medicine, London, United Kingdom; 3 Department of Control of Neglected Tropical Diseases, World Health Organization, Geneva, Switzerland; Federal University of Ceará, Fortaleza, Brazil, BRAZIL

## Abstract

**Background:**

Trachoma is targeted for global elimination as a public health problem by 2030. Understanding individual, household, or community-associated factors that may lead to continued transmission or risk of recrudescence in areas where elimination has previously been achieved, is essential in reaching and maintaining trachoma elimination. We aimed to identify climatic, demographic, environmental, infrastructural, and socioeconomic factors associated in the literature with trachoma at community-level and assess the strength of their association with trachoma. Because of the potential power of geospatial analysis to delineate the variables most strongly associated with differences in trachoma prevalence, we then looked in detail at geospatial analysis methods used in previous trachoma studies.

**Methods:**

We conducted a systematic literature review using five databases: Medline, Embase, Global Health, Dissertations & Theses Global, and Web of Science, including publications from January 1950 to January 2021. The review protocol was prospectively registered with PROSPERO (CRD42020191718).

**Results:**

Of 35 eligible studies, 29 included 59 different trachoma-associated factors, with eight studies also including spatial analysis methods. Six studies included spatial analysis methods only. Higher trachomatous inflammation—follicular (TF) prevalence was associated with areas that: had lower mean annual precipitation, lower mean annual temperatures, and lower altitudes; were rural, were less accessible, had fewer medical services, had fewer schools; and had lower access to water and sanitation. Higher trachomatous trichiasis (TT) prevalence was associated with higher aridity index and increased distance to stable nightlights. Of the 14 studies that included spatial methods, 11 used exploratory spatial data analysis methods, three used interpolation methods, and seven used spatial modelling methods.

**Conclusion:**

Researchers and decision-makers should consider the inclusion and potential influence of trachoma-associated factors as part of both research activities and programmatic priorities. The use of geospatial methods in trachoma studies remains limited but offers the potential to define disease hotspots and areas of potential recrudescence to inform local, national, and global programmatic needs.

## Author summary

The ambitious target to eliminate trachoma as a public health problem has led to impressive strides in reducing the disease burden worldwide, with the implementation of the World Health Organization (WHO)-endorsed surgery, antibiotics, facial cleanliness and environmental improvement (SAFE) strategy. However, some areas have struggled to reach the elimination threshold after the prescribed number of antibiotic mass drug administration rounds, and some areas have had evidence of trachoma recrudescence after previously having reached the elimination threshold. This systematic review assessed climatic, demographic, environmental, infrastructural, and socioeconomic factors associated with trachoma to reveal which covariates are associated with ongoing or renewed trachoma transmission. We also explored how geospatial analysis, which could help identify areas with ongoing trachoma transmission or heightened risk of recrudescence, has been used in previous trachoma studies. Thirty-five studies met the inclusion criteria for the review. Results indicated that researchers and decision-makers should consider the inclusion and potential influence of precipitation, temperature, and altitude along with variables related to ruralness, accessibility, access to medical services and schools, and community-level water and sanitation coverage, as part of both research activities and programmatic priorities for trachoma elimination.

## 1 Background

Trachoma, the leading infectious cause of blindness, is targeted for global elimination as a public health problem by 2030. Since 1993, integrated interventions to achieve this have been summarised using the acronym ’SAFE’: **s**urgery for trachomatous trichiasis (TT, the potentially blinding stage of the disease); **a**ntibiotics to clear the infection (distributed using mass drug administration, MDA); **f**acial cleanliness and **e**nvironmental improvement to limit transmission [1]. The WHO simplified grading system [2,3] is the gold standard for identifying five specific clinical signs of trachoma in population-based prevalence surveys [4]: it does not require an eye specialist and can be performed by well-trained personnel. Laboratory diagnosis of ocular *Chlamydia trachomatis* (Ct) infection is possible, although infection testing is not currently used for programmatic or elimination validation purposes [4]. The elimination validation indicators are both clinical and programmatic: prevalence of trachomatous inflammation—follicular in 1–9-year-olds (TF1–9) <5% in each formerly endemic district, a prevalence of TT "unknown to the health system" in ≥15 year-olds <0.2% in each formerly endemic district (TT unknown to the health system excludes recurrent cases, cases who have refused management, and those who are scheduled for surgery)[5], and the existence of a system able to identify and manage incident TT cases, using defined strategies, with evidence of appropriate financial resources to implement those strategies [6].

The ambitious target to eliminate trachoma as a public health problem has led to impressive strides in reducing the disease burden. The SAFE strategy has been very effective in widely disparate contexts, with the prevalence of TF and TT falling below the elimination thresholds in many districts [7,8]. However, some areas have had challenges in reaching the elimination threshold after the prescribed number of MDA rounds, and some areas have had evidence of recrudescence. Recrudescence is the return of a prevalence of ≥5% of TF1–9 in an area that previously reduced its prevalence below 5% [9–11]. Districts that have a TF1–9 prevalence of <5% may be at risk of recrudescence post-validation and may need continued surveillance to ensure TF prevalence does not exceed 5% in the future [6]. Understanding individual, household, or community-associated factors that are associated with higher prevalence may help decision-makers to tailor strategies for reaching and maintaining the global elimination of trachoma.

Key trachoma transmission routes are thought to include [12]: direct close contact spread from eye to eye through ocular or nasal secretions, indirect spread by fomites [13], and transmission by eye-seeking flies (e.g. *Musca sorbens*) [14,15]. Trachoma is a highly focal disease, found to cluster in communities and within households [12]. Previous reviews and more recent studies demonstrate that trachoma is associated with individual- and household-level factors including inadequate access to water which can affect hygiene practices (lack of cleaning face with soap) [16], poor sanitation (the specific fly vector species preferentially breeds in human faeces left exposed on the soil) [17], and household crowding [4,12,18]. However, trachoma is a community disease usually managed at district level. In this review, therefore, we systematically review associated factors at the community or district level.

Given the challenges in reaching elimination in some areas, there is a need for new or enhanced approaches for identifying areas of ongoing trachoma transmission or heightened risk of recrudescence. Furthermore, in an increasingly resource-constrained global health programme environment, tools are needed that can leverage existing data to answer programmatic and research questions. One such approach is geostatistical modelling which can maximise the use of existing trachoma data to understand trachoma prevalence in areas of ongoing transmission or areas that have experienced, or are at risk of, recrudescence. This type of targeted approach, along with purposive adaptive sampling, is one proposed way to meet the elimination end-game challenges [19]. A key input to geostatistical modelling approaches is geospatial covariates of associated factors for the disease.

This systematic review assesses climatic, demographic, environmental, infrastructural, and socioeconomic factors associated with trachoma to provide up-to-date information on covariates to include in future geospatial analyses. It is based in part on the approach used by a previously-published review [20]. The primary geographic focus, or unit of analysis, will be the community, village, survey cluster, or district, collectively referred to as "community" henceforth. The research question for this review, using the population, interventions/exposures, comparators, outcomes and study designs (PICOS) framework [21], is as follows:

- Which climatic, demographic, environmental, infrastructural, socioeconomic, geographic, and spatial proximity factors are associated with trachoma prevalence, measured by TF, TT, or ocular Ct infection in communities where trachoma prevalence has been assessed within trachoma-endemic countries?

The main objectives of the review are to:

- Identify factors associated with trachoma at the community level in the literature and assess their potential impact on trachoma.
- Identify geospatial analysis methods used in previous trachoma studies.

## 2 Methods

The review protocol was registered with PROSPERO in July 2020 (CRD42020191718) (S1 Text). Before submission, the protocol was reviewed by a reference librarian at the London School for Hygiene & Tropical Medicine (LSHTM, June 2020) using the PRESS guidelines [22]. This manuscript was written following the Preferred Reporting Items for Systematic reviews and Meta-Analysis (PRISMA) 2020 statement guidelines and checklist [23] (S2 Text).

### 2.1 Eligibility criteria

We included peer-reviewed studies and published theses reporting on trachoma prevalence (cross-sectional, case-control, and cohort study designs) that described associations between trachoma prevalence and climatic, demographic, environmental, infrastructural, socioeconomic, geographic, and spatial proximity factors measured at community level. Full Inclusion and exclusion criteria are listed in Table 1.

**Table 1:** Inclusion and exclusion criteria used for selecting studies for the full review.

MEASURE	INCLUSION	EXCLUSION
**POPULATION**	Human subjects in any community where trachoma prevalence has been assessed	Non-human participants, genomic, cellular, or another laboratory-based research
**INTERVENTION/EXPOSURE**	Climatic, demographic, environmental, infrastructural, socioeconomic, geographic, or spatial proximity factors, measured at community level	Papers reporting on factors measured at the individual or household level
**OUTCOME**	Trachoma prevalence measured by active trachoma TF*, TT**or ocular Ct*** infection (measured by a nucleic acid amplification-based test)	Papers reporting on conditions not related to the eyes, including sexually transmitted infections and urogenital chlamydia. Research that does not include prevalence results such as MDA^ coverage or the impact of treatment on other disease outcomes
**TYPE OF STUDY**	Peer-reviewed publication, thesis, or conference/poster abstract, reporting primary data collection results or secondary data analysis	Review articles, guidelines, mathematical modelling studies, or opinion pieces
**DATE**	Published from 1 January 1950–31 January 2021	Published before 1950 or after 1 February 2021
**LANGUAGE**	English, French, Spanish, or Portuguese^^	Any language other than English, French, Spanish, or Portuguese

* TF (trachomatous inflammation—follicular)

**TT (trachomatous trichiasis)

***Ct (*Chlamydia trachomatis)*

^MDA (Mass Drug Administration)

^^These languages were chosen based on the language skills of the reviewing individuals.

### 2.2 Information sources and search strategy

We conducted the systematic search in five databases: Medline (US Medical Library/Ovid), Embase (Elsevier/Ovid), Global Health (CABI, Ovid), Dissertations & Theses Global, and Web of Science (Clarivate Analytics). Two search rounds were conducted, by the lead researcher (Burgert), in all five databases, first on 2 July 2020 for the dates January 1950 through June 2020 and later updated on 3 February 2021 to include July 2020 through January 2021. The search terms were a mix of medical subject header terms (MeSH) and free text keywords; the specific terms used were adapted for each database. The final search terms for each database are summarised in S3 Text.

The basic structure of the search was split into two main components joined by AND:

- Component 1: trachoma terms (e.g., trachoma, trichiasis, ocular chlamydia) joined by OR
- Component 2: associated factor terms joined by OR
  ○ Climatic terms (e.g., rain, temperature, sunshine, aridity)
  ○ Demographic terms (e.g., population density, crowding, migration)
  ○ Environmental terms (e.g., altitude, soil, land cover)
  ○ Infrastructure terms (e.g., rural/remoteness, access, water, sanitation)
  ○ Socioeconomic terms (e.g., housing, crowding, population, poverty)
  ○ Geographic/spatial terms (e.g., GIS, raster, spatial clustering)

### 2.3 Selection process

All records were assembled, and duplicates were removed using Endnote Version X8.2. All retained non-duplicate studies were reviewed in a two-step process. Each study title/abstract was reviewed in step one and given a primary reason for exclusion/inclusion by two researchers working independently (Burgert and either Adams or Mingkwan), recorded in a Microsoft Excel Version 2012 [24] table. The reasons for exclusion were:

1. Missing abstract or full text not found
2. Language other than English, French, Spanish, or Portuguese
3. Non-human research (genomic, cellular, or other laboratory-based)
4. Systematic review, guidelines, mathematical modelling study, or opinion piece
5. Study of condition(s) not related to the eyes, including sexually transmitted infections
6. Report on trachoma research not including prevalence results, such as MDA coverage or impact of treatment on other disease outcomes only
7. Report on individual or household trachoma prevalence alone, only individual- or household- associated risk factors or no geospatial methods used

In step two, the abstracts of all studies meeting the population, outcome, type of study, date, and language criteria, regardless of the inclusion of selected interventions (exclusion criteria 6 and 7 plus all included studies), were reviewed by three researchers independently (Burgert, Adams, and Mingkwan). If any reviewer felt that any abstract met the inclusion criteria, the study was included in the next stage. A full-text review of studies for final inclusion was performed by two researchers independently (Burgert and either Adams or Mingkwan). The three research team members discussed any disagreements, and a consensus agreement was made regarding inclusion. Furthermore, studies were excluded if an overall relationship/trend (e.g., percentage, odds ratio [OR]) could not be synthesised between the associated factor described and the outcome.

### 2.4 Data extraction

The extraction of information for included studies was performed by one researcher (Burgert) and reviewed by one researcher (Adams). Extracted data were tabulated in Microsoft Excel Version 2012 [24]. The primary data items collected for this review were trachoma outcome measures, associated factor variables, how factors were measured, and data source of factors; along with study year, population, sampling, and design. The primary outcome domains recorded were the signs of TF and TT from the WHO simplified grading system [2,3] or ocular Ct infection (measured by a nucleic acid amplification-based test). Though the WHO simplified grading system was not officially published until 1987, studies measuring the primary outcomes of TF or TT using similar or comparable grading methods prior to 1987 were included [2]. Trachoma outcomes were recorded as measured in the study, such as TF or TT, including any specific age ranges. Analysis type, univariable (outcome and associated factor only) or multivariable (outcomes and associated factor plus other confounding factors such as age and sex/gender), were recorded for all studies.

Specific associated factor variables measured in the studies were combined into variable groups, e.g., precipitation (variable group) encompasses mean annual precipitation (specific variable) and monthly rain days (specific variable). Variable groups were further combined into variable categories, e.g., climate (variable category) includes temperature (variable group) and precipitation (variable group). Five variable categories were used: climate, demography, environment, infrastructure, and socioeconomic status. For studies that included multiple outcome measures or multiple associated factors, an individual row for each combination of the outcome, associated factors, and analysis type was included in the Excel spreadsheet. For example, studies reporting both a univariable and multivariable result for an outcome and associated factor pair had both results recorded. Thus, a paper reporting univariable and multivariable results on TF and TT with associated factors of altitude and temperature would have a total of eight rows in the spreadsheet. The data source for each associated factor was also recorded, including if it was an external geospatial raster, an external georeferenced data source, or collected/measured within the same survey as the trachoma outcome data. Study results were recorded as ORs or percentages, along with any reported confidence intervals (CI) and p-values, and any controlling factors from the analysis. The statistical significance of results, either noted as p-values or non-overlapping confidence intervals, was also documented.

### 2.5 Quality assessment

Study quality was assessed using the STROBE checklist for observational studies (cohort, case-control, and cross-sectional studies) [25]. Specific areas considered to assess overall quality were:

1. Survey type: population-based, cohort, or school-based.
2. Survey population and level of representation: population target, population examined, number of survey clusters, and number of evaluation units/districts or other levels of representation for the sample.
3. Use of WHO survey guidance [26,27] or similar for survey sampling methods; although many studies might have taken place earlier than the guidance was released, all studies were measured against the current WHO guidance.
4. Survey focus: trachoma only or multiple diseases/outcome measures.
5. Method of trachoma identification: using WHO simplified grading system to measure trachoma outcomes [2,3] and ophthalmic magnifying loupes.

An overall quality assessment was given for each study. A high assessment was given to studies that met the current WHO guidance, including being population-based, use of the correct study population for outcome of interest, inclusion of 20–30 survey clusters per evaluation unit, and positioning of trachoma as a primary outcome of the survey. A medium assessment was given to studies that met most but not all the criteria for a high assessment, while a low-quality assessment was for cohort or school-based studies or those in which the population-based sample was not sufficient to be representative of a given evaluation unit.

### 2.6 Data synthesis and certainty assessment methods

Due to the heterogeneity of study methods and differences in how associated factors were reported (varying results categories, e.g., altitude measured as a continuous or categorical variable), a meta-analysis was not possible and instead a synthesis of results was created. Factors associated with the outcome were summarised to indicate trend direction (e.g., increasing altitude) and impact on outcome (e.g., lower TF prevalence). For factors that were categorical by nature, such as urban versus rural areas, one category was selected and assessed by comparing how its presence/absence impacted the outcome (e.g., higher TF prevalence).

Table 2 describes the three results categories and the types of results recorded in each. Studies with both univariable and multivariable results for the same outcome and associated factor pair were combined to report only one result for the included study. If the univariable and multivariable results differed within the same study, the outcome was listed as "mixed." Results were also listed as mixed if the data indicated differing trends for the same factor at different categorical levels. Statistical significance of results was summarised from information provided in studies such as p-values or non-overlapping CIs; if any reported result was statistically significant, the overall result for that factor in that study was reported as being statistically significant. All associated factors with three or more synthesised measurement results were considered to have sufficient information to conclude the effect of associated factors with at least some certainty in the results. These results were summarised separately for TF and TT and included in an evidence map for ease of interpretation. Associated factors with only one or two reported results were considered to have insufficient information on which to draw any overall conclusions.

**Table 2:** Data synthesis measurement summary.

As Factor Increases or As Factor is Present, there is…	Lower prevalence	Mixed both higher and lower prevalence	Higher prevalence
**Result types**	1) Only a univariable or multivariable result reported; all categories have the same trend 2) Both univariable and multivariable results reported for the same factor; all results categories show the same trend	1) Only a univariable or multivariable result reported; categories have different trends and no specific trend direction 2) Both univariable and multivariable results reported for the same factor and result categories have different trends and no specific trend direction 3) Univariable and multivariable results reported are NOT the same and have differing trend directions	1) Only a univariable or multivariable result reported; all categories have the same trend 2) Both univariable and multivariable results reported for the same factor; all results categories show the same trend

## 3 Results

### 3.1 Overview of included studies

**Fig 1:**
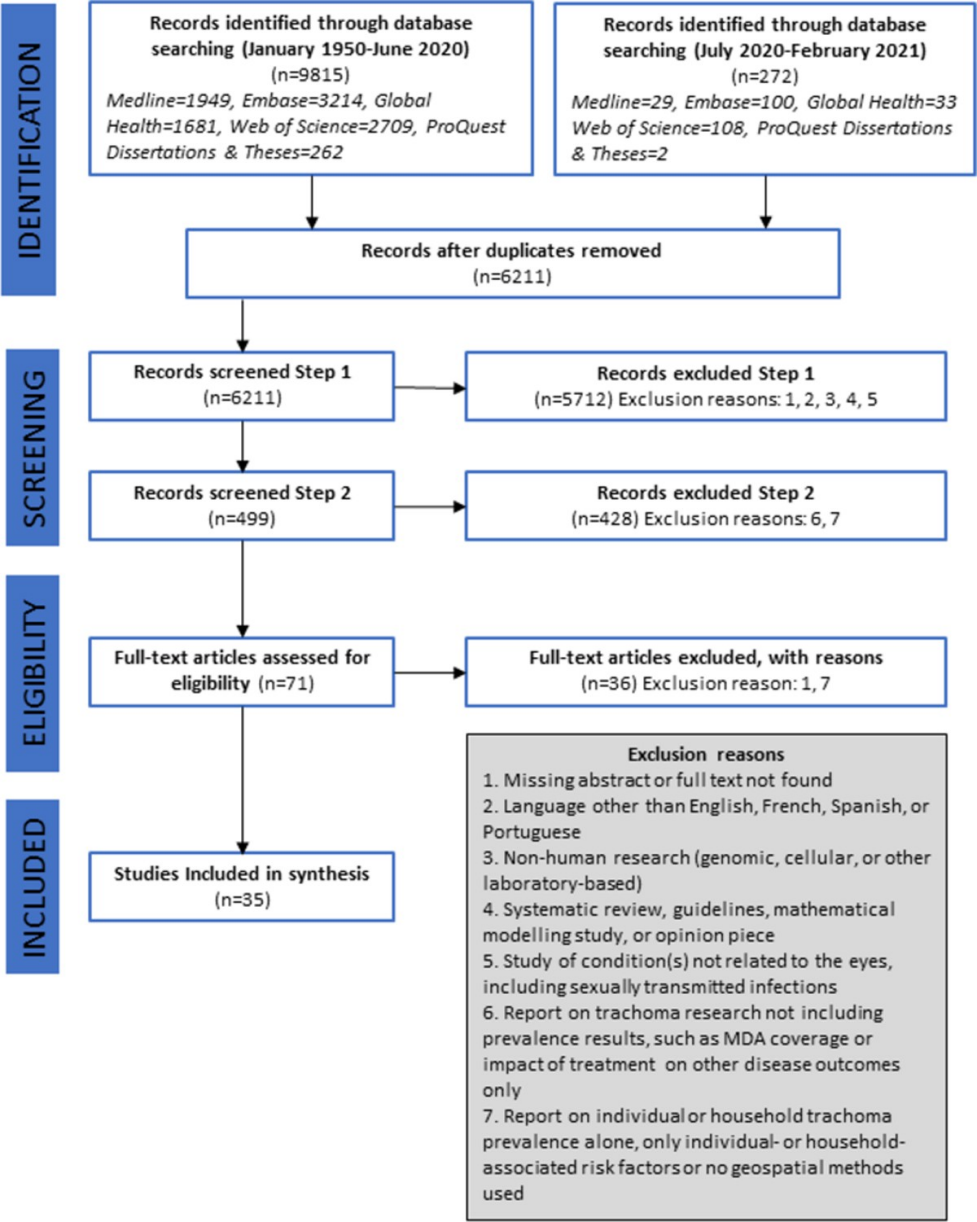
Study inclusion flowchart.

A total of 10,087 records were identified from the database search; of these, 6,211 were unique records. All 6,211 were screened, resulting in eligibility being assessed on 71 full-text records, of which 35 studies were retained (Fig 1). Of the 35 included studies [28–62], 29 included trachoma associated factors, of which eight also included spatial analysis methods [30,33,34,37,47,58,60,61]; six studies included spatial analysis methods only [32,35,46,49,54,56] (Table 3).

**Table 3:** Summary of selected studies by survey type, study quality measures, trachoma outcome, and analysis type.

Reference	Survey Start Year	Study Quality Measures															Trachoma Outcome (Percent)			Analysis Type		Country
		Survey Type			Survey Population & Representation				Survey Focus		Use of guidelines			Trachoma Identifica- tion		Overall Quality Score						
		Population Based	Cohort/Other	School	Population Target	Population Examined	Number of Survey Clusters/ Schools	Evaluation unit(s)/ District(s)	Trachoma only	Multiple	WHO	Similar to WHO	Other	WHO Grading	Use of Loupes		TF	TT	Other	Factor Association	Geospatial	
**Adera, T. 2016 [28]**	2013	X			All	121,397	1,047	40	X		X			X		High	2.3–48.5			X		Ethiopia
**Alemayehu, W 2005 [29]**	2000	X			1 to 6 years	2,788	25	1		X			X	X	X	Medium	56.5			X		Ethiopia
**Altherr, F. 2019 [30]**	2011	X			All	202,312	1,558	152	X			X		X		High	25.1			X	X	Ethiopia
**Baggaley, R. 2006 [31]**	2002	X			1 to 9 years	12,415	64	1	X				X	X		Medium	9.1			X		Tanzania
**Bailey, R. 1989 [32]**	NR		X		All	911	1	1	X				X	X	X	Low	12.2				X	The Gambia
**Bero, B. 2016 [33]**	2012	X			All	127,357	2,037	79	X		X			X		High	9.6–60	0–12		X	X	Ethiopia
**Clements, A. 2010 [34]**	2001	X			1 to 9 years	6,345	112	5	X			X		X		High	2.2–77.6			X	X	South Sudan
**Edwards, T. 2012 [35]**	2010	X			1 to 9 years	2,406	40	1	X			X		X		High	70.5				X	South Sudan
**Elshafie, B. 2016 [36]**	2015	X			All	73,489	908	32	X		X			X		High	0–18.7	0–1.2		X		Sudan
**Flueckiger, R. 2019 [37]**	2012	X			1 to 9 years & 15+ years	810,975 & 964,910	15,051	624	X		X			X		High		0.2–2.2		X	X	Multiple^
**Garn, J. 2018 [38]**	2012	X			1 to 9 years	884,850	13,451	NR	X		X			X		High	1–19.5			X		Multiple^^
**Hagi, M. 2010 [39]**	1996	X			< 10 years	14,627	210	7 regions	X			X		X		Medium	35.0			X		Mali
**Haileselassie, T. 2007 [40]**	2001	X			1 to 10 years	1,169	9	1	X				X	X		Low	41.3			X		Ethiopia
**Jie, Y. 2008 [41]**	2001		X		40+ years	4,411	7	1 region		X			X	X		Low			9.6*	X		China
**Katz, J. 1996 [42]**	1990	X			24–76 months	836	40	1		X			X	X	X	Medium	23.6			X		Nepal
**Ketema, K. 2012 [43]**	2012	X			1 to 9 years	792	5	1	X				X	X	X	Low	17.2			X		Ethiopia
**Khandekar, R. 2006 [44]**	2001	X			35+ years	43,677	~390	12	X				X	X		Medium		1.8–13.7		X		Vietnam
**Koukounari, A. 2011 [45]**	2007			X	7 to 11 years	3,324	21	11 regions	X				X	X		Low	0–35.65			X		Burkina Faso
**Last, A. 2017 [46]**	2005	X			All	1,507	38	4 islands	X					X	X	Medium	NR		NR**		X	Guinea Bissau
**Luna, E. 1992 [47]**	1986	X			All	2,908	4 strata	1	X				X	X	X	Low	1.8			X	X	Brazil
**Luna, E. 2016 [48]**	2002			X	1–4 grade children	185,862	NR	27 states	X				X	X	X	Low	1.5–9.0			X		Brazil
**Macharelli, C. 2013 [49]**	2005			X	6 to 15 years	1,054	12	1	X				X	NR		Low	6.3				X	Brazil
**Medina, N. 2011 [50]**	2003			X	1–4 grade children	6,986	NR	1 region	X				X	X	X	Low	4.5			X		Brazil
**Mpyet, C. 2010 [51]**	2006	X			1 to 5 years	639	27	1	X			X		X	X	Medium	35.7			X		Nigeria
**Ngondi, J. 2008 [52]**	2006	X			1 to 9 years & 15+ years	5,427 1 & 9,098	160	10 zones	X			X		X		Medium	24.5	5.9		X		Ethiopia
**Oswald, W. 2017 [53]**	2011	X			1 to 9 years	62,869	1,510	6 zones	X			X		X	X	High	29			X		Ethiopia
**Polack, S. 2005 [54]**	2000		X		All	956	1	1	X				X	X		Low	20.3				X	Tanzania
**Sahlu, T. 1992 [55]**	1988	X			All	1,222	10	1	X				X	X	X	Low	26.8			X		Ethiopia
**Schellini, A. 2010 [56]**	2005			X	1–4 grade children	2,692	NR	1	X				X	X	X	Low	2.9				X	Brazil
**Schemann, J. 2002 [57]**	1996	X			< 10 years	15,187	210	7 regions	X			X		X		Medium	34.9			X		Mali
**Schemann, J. 2007 [58]**	1996	X			<10 years & 15+ year women	14,656 & 11, 086	210	7 regions	X			X		X		Medium	34.9	23.7		X	X	Mali
**Silva, E. 2020 [59]**	2018			X	7 to 16 years	478	NR	1	X				X	X	X	Low	34.9			X		Brazil
**Smith, J. L. 2014 [60]**	2004	X			1 to 9 years	21,003	438	17	X			X		X		Medium	18.8			X	X	Kenya
**Smith, J. L. 2015 [61]**	2005	X			40+ years	13,543	304	national		X		x		X		Medium		1.45		X	X	Nigeria
**West, S. 1991 [62]**	1986	X			1 to 7 years	4,000	20	1	X				X	X	X	Medium	60			X		Tanzania

WHO: World Health Organization, TF: Trachomatous inflammation—follicular, TT: Trachomatous trichiasis, NR: Not Reported, X*: Measured as "Any trachomatous change"; X**: Ocular *Chlamydia trachomatis* infection; ^Multiple countries: Benin, Côte d’Ivoire, Democratic Republic of the Congo Ethiopia, Guinea, Malawi, Mozambique, Nigeria, Sudan, Uganda; ^^Multiple countries: Benin, Côte d’Ivoire, Democratic Republic of the Congo Egypt, Ethiopia, Guinea, Malawi, Mozambique, Nigeria, Lao People’s Democratic Republic, Solomon Islands, Vanuatu, Yemen

All but one study, which was published in a dissertation [60], were published in peer-reviewed journals. The publication period for included studies ranged from 1989 to 2020. Data collection for included studies was reported as early as 1986 [47,62], with the most recent data collection occurring in 2018 [59], with one study published in 1989 not reporting data collection dates [32]. Of the 35 studies, there were 33 distinct data collection activities. Three studies used the same data collected during the "Mali National Trachoma Survey" but had different objectives or outcomes and performed distinct data analysis activities with no overlapping variables [39,57,58]. The 35 studies included data from 26 countries. Most studies focused on a single country, while two involved a multi-country analysis [37,38]. The data used in the multi-country data analysis activities either did not include data used in the other 34 studies and/or had no overlapping variables with those other studies. TF prevalence ranged from 0% to 77% across 30 studies, while TT prevalence ranged from 0% to 23% across seven studies; one study did not report trachoma prevalence as a percent of the population. In most cases, the associated factor analysis did not disaggregate according to survey prevalence, treatment status, or past survey prevalence.

### 3.2 Study quality

Study quality results are summarised in Table 3. Most manuscripts reported data collected in a cross-sectional population-based survey, while two manuscripts reported a cohort study design [45,54]. The cross-sectional studies were chiefly population-based surveys with a two-stage sampling design for survey clusters (villages) and then households. In contrast, most of the studies in Brazil (five of six) [48–50,56,59] and one study in Burkina Faso were school-based surveys [45]. All but four studies were surveys solely focused on trachoma. Of the four multi-disease surveys, one had the trachoma component embedded as part of a Vitamin A assessment [42], two were part of a country adult blindness survey [41,61], and one was a joint trachoma and schistosomiasis survey [45]. Of the 25 population-based survey studies, 16 followed WHO survey guidance or similar; of these, five were conducted with support from the Global Trachoma Mapping Project (GTMP), which provided gold standard support for surveys conforming to WHO guidance from 2012–2016 [63]. The remaining nine population-based surveys measuring trachoma prevalence were stand-alone studies that did not specify the basis for their sampling strategy. The study population varied in the studies but was most commonly 1–9-year-olds for TF and ≥15 year-olds for TT. The majority of studies (32 of 35) reported specifically using the WHO simplified grading system for trachoma. One study did not specify the trachoma identification method used [49]. The use of 2.5× magnifying loupes was mentioned in 12 studies, with a further two using 3.5× or 4× loupes. All other studies, including the five GTMP studies, made no specific mention of the use of magnifying loupes, although it was standard practice for GTMP surveys. Overall quality for the 35 studies was evenly divided across the three categories: high (10), medium (13), and low (13).

### 3.3 Associated factors and trachoma studies

Twenty-nine studies included trachoma outcomes with associated factors (Table 4). The most common trachoma outcome studied was TF (25 studies) [28–31,34,38–43,45,47,48,50,51,53,55,59–62], while TT was analysed in seven studies [33,36,37,44,52,58,61,62]. Only four studies included analysis for both TF and TT outcomes [33,36,52,58]. One study in adults from China included "any trachomatous change" as their primary outcome; the one associated factor identified in this study (ruralness) was not included in the synthesised results [41]. Seven studies included only univariable analysis [29,44,47,48,51,55], eight only multivariable results [30,31,34,37–39,41,53], and 14 reported univariable and multivariable results [28,33,36,40,42,43,45,50,52,57–61]. Thus, the recording of results included 196 unique study, outcome, variable, and analysis type (univariable/multivariable) combinations.

**Table 4:** Associated factor studies by trachoma outcome, analysis type, and variable category.

Reference		Adera, T. 2016 [28]	Alemayehu, W 2005 [29]	Altherr, F. 2019 [30]	Baggaley, R. 2006 [31]	Bero, B. 2016 [33]	Clements, A. 2010 [34]	Elshafie, B. 2016 [36]	Flueckiger, R. 2019 [37]	Garn, J. 2018 [38]	Hagi, M. 2010 [39]	Haileselassie, T. 2007 [40]	Jie, Y. 2008 [41]	Katz, J. 1996 [42]	Ketema, K. 2012 [43]	Khandekar, R. 2006 [44]	Koukounari, A. 2011 [45]	Luna, E. 1992 [47]	Luna, E. 2016 [48]	Medina, N. 2011 [50]	Mpyet, C. 2010 [51]	Ngondi, J. 2008 [52]	Oswald, W. 2017 [53]	Sahlu, T. 1992 [55]	Schemann, J. 2002 [57]	Schemann, J. 2007 [58]	Silva, E. 2020 [59]	Smith, J. L. 2014 [60]	Smith, J. L. 2015 [61]	West, S. 1991 [62]
**Trachoma Outcome**	**TF**	X	X	X	X	X	X	X		X	X	X		X	X		X	X	X	X	X	X	X	X	X	X	X	X		X
	**TT**					X		X	X							X						X				X			X	
	**Other**												X*																	
**Analysis type**	**Univariable**	X	X			X		X				X		X	X	X	X	X	X	X	X	X		X	X	X	X	X	X	X
	**Multivariable**	X		X	X	X	X	X	X	X	X	X	X	X	X		X			X		X	X		X	X	X	X	X	
**Number of variables by variable category**	**Climate**	3		2		2	1	2	1		4						5									3		5	3	
	**Demographic**								1				1					1	2	2					1		1	3	2	
	**Environment**	1	1	1	1	1	3		1			2			1	1	1					1		1		3		5	4	
	**Infrastructure**			2						2				3							2		1		3			8		3
	**Socioeconomic**																								2			4	1	
	**Total**	4	1	5	1	3	4	2	3	2	4	2	1	3	1	1	6	1	2	2	2	1	1	1	6	6	1	25	10	3

TF: Trachomatous inflammation—follicular, TT: Trachomatous trichiasis, X*: Measured as "Any trachomatous change"

Overall, 59 different associated factors (S4 Text) were included in the 29 included studies. The infrastructure variable category had the most (23) associated factor variables across all the studies. Table 4 summarises the number of associated factors by variable category per study. The number of unique associated factors included in any individual study ranged from 1 to 25; ten studies included only one factor, while 17 had between two to six factors. The majority of studies (16) had at least one factor in the environment category.

Results are reported for each of the five variable categories, focusing on those associated factors included in three or more studies. Synthesised results for TF and TT all associated factors are included in S1 Table, while full results for each study are in S2 Table. Statistical significance of results is also presented as a binary outcome: "yes" if any of the reported results were significant and "no" if all results were not significant.

The source of covariate data included in the 29 studies with associated factors varied from geospatial raster datasets, survey-derived, government data or other survey data, meteorological station data, and direct global positioning system (GPS) reading. S4 Text summarises the sources of data for each variable. Survey-derived covariates include information collected as part of the same survey being analysed; this included many infrastructure variables such as water and sanitation coverage, presence of medical services, or paved roads. Other survey-derived variables included rural/urban classification. Government data sources or other survey data included information on school attendance and poverty measures; these data were linked to survey location by administrative unit information. GPS data collected during surveys were used for latitude, longitude, and in some cases, altitude measures.

### 3.3.1 Associated factor trends: Climate

**Fig 2:**
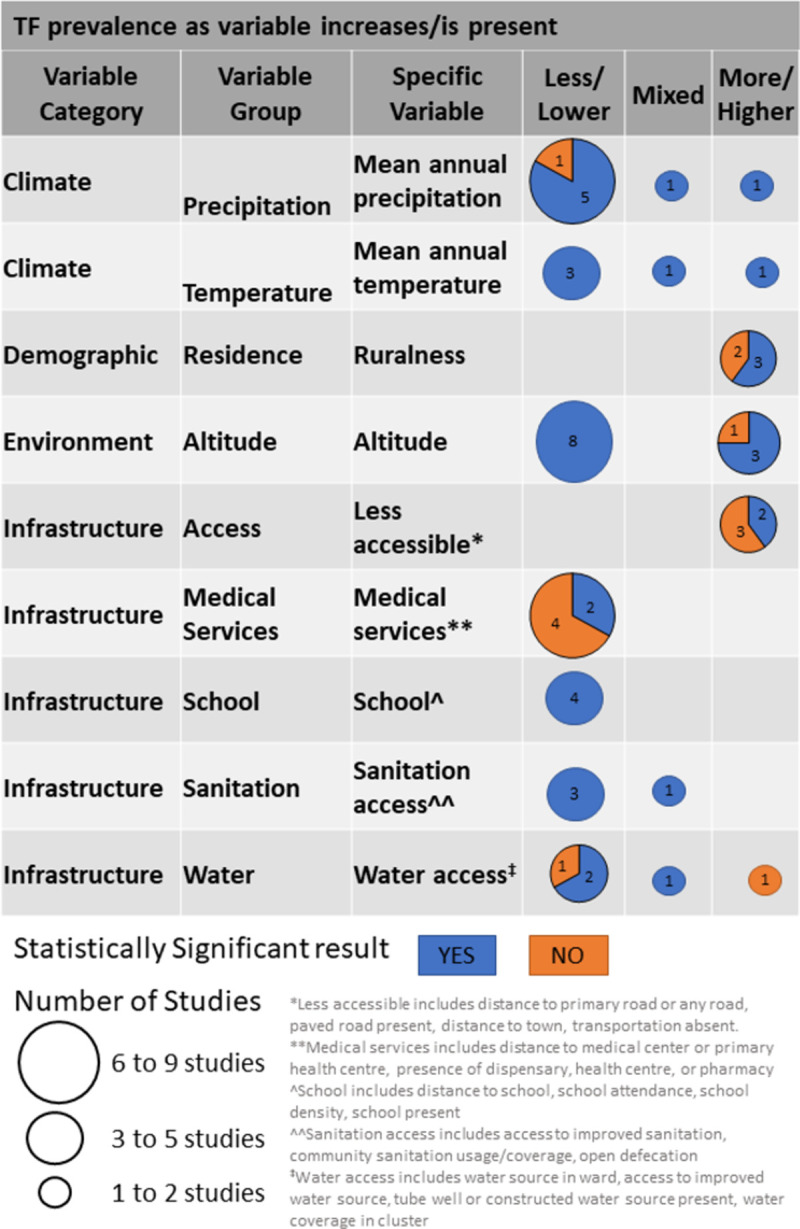
Associated factor trends for TF.

The climate variable category included 14 unique variables used in studies examining TF. Only the specific variables for mean annual precipitation and mean annual temperature were used in three or more studies. The overall trend was that as mean annual precipitation increases, TF prevalence is lower; six of eight studies supported this conclusion (Fig 2) [30,33,34,36,58,60], of which five were statistically significant. The results for aridity index, relative humidity, air pressure, sunshine fraction, and monthly rainfall days were also in-line with this finding but had two or fewer studies using these variables (S1 Table). Mean annual temperature was included in five statistically significant analyses, with three indicating lower TF prevalence as temperature increases [30,39,45]. There were seven other temperature measures included across seven studies [28,33,36,39,45,58,60], but in all cases, two or fewer per specific variable (S1 Table).

**Fig 3:**
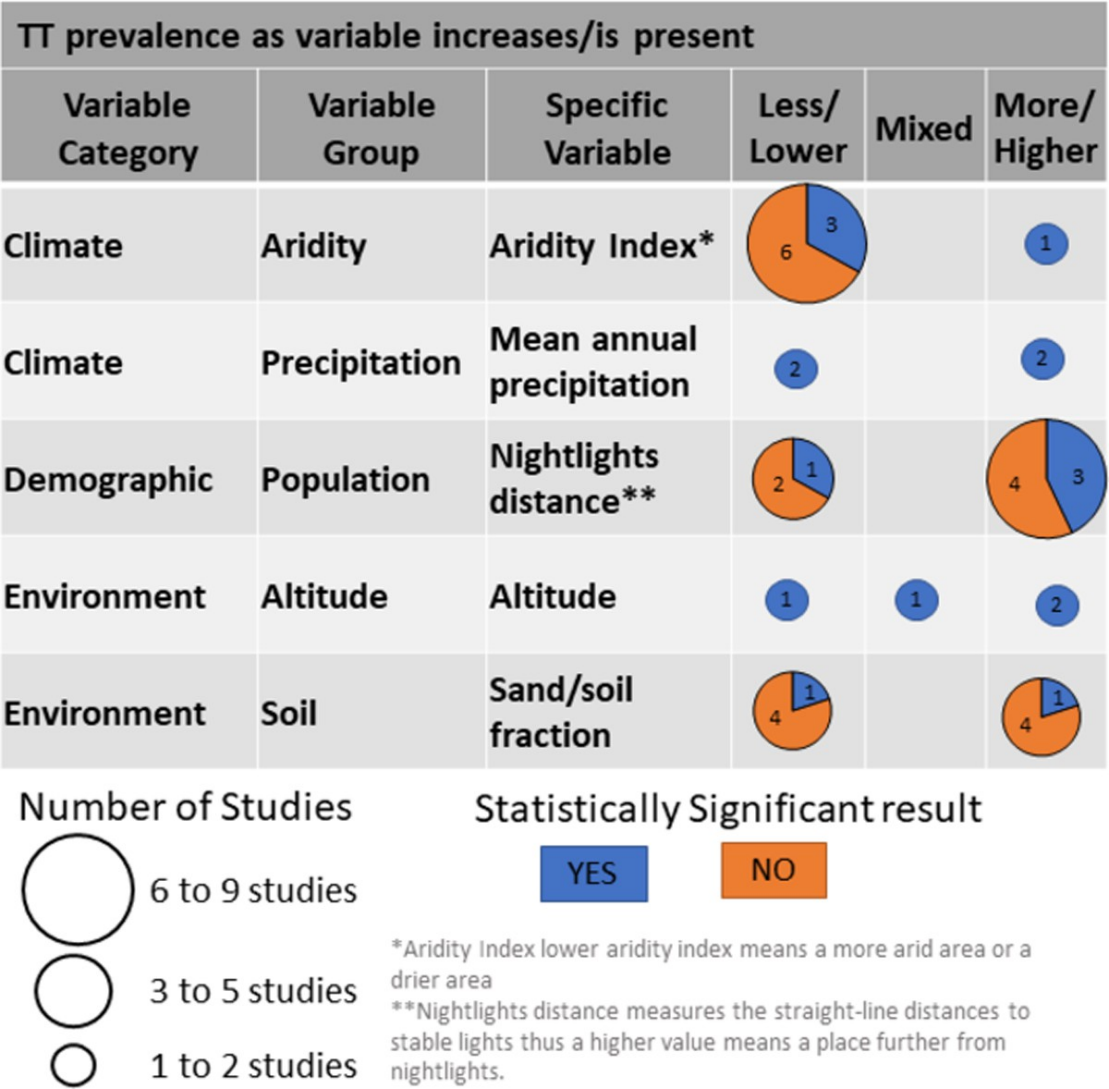
Associated factor trends for TT.

For studies looking at TT outcomes, aridity (lower aridity index means more arid area/drier area) and mean annual precipitation had enough results for synthesis. Data showed that areas with less aridity had lower TT prevalence (Fig 3) (nine of ten analyses, though only three of nine were statistically significant). Of note, all these analyses came from Flueckiger *et al*. 2019 [37], which analysed data from ten countries. Mean annual precipitation was included in four studies, but results were inconclusive [33,36,58,61]. All other TT results for climatic factors had two or fewer studies using the same variable, and thus no conclusions could be drawn.

### 3.3.2 Associated factor trends: Demographic

Within the demographic variable category, five variables were used across six studies examining TF. Areas considered to be "rural", as classified by the study survey design, had higher TF prevalence than "urban" areas (Fig 2) (four of five results from Brazil studies and three of five statistically significant) [47,48,50,59,60]. All other demographic variables did not have three or more studies that included the same measurement. The variable group that measured population density and size of the population in a village showed a trend of areas with decreasing population having higher TF prevalence [48,57,60].

Distance to nightlights, a proxy measure for population density or development, was used in a ten-country TT analysis [37]. Areas with fewer nightlights had higher TT prevalence (seven of ten, and three of seven were statistically significant) (Fig 3).

### 3.3.3 Associated factor trends: Environment

Within the environment category, five main variable groups were included in the TF studies. Altitude was by far the most common variable, being included in twelve analyses. Eight studies showed higher altitude areas had lower TF prevalence, and all associations were statistically significant (Fig 2) [28,29,31,33,40,43,55,58]. Ethiopia had eight of twelve results related to altitude [28–30,33,40,43,52,55], including six of eight in the "increasing altitude, lower TF prevalence" group. None of the other environment variables had three or more studies that included the same measurement.

For TT, six main variables were included in the studies. Altitude was included in four analyses [33,52,58,61] and showed mixed results with no clear trends (Fig 3). The sand/soil fraction, an indicator of dry or dusty earth, was equally split between higher or lower TT prevalence across ten countries [37].

### 3.3.4 Associated factor trends: Infrastructure

The infrastructure category had 23 unique variables. None was used in more than one analysis in studies with TF as the main outcome; however, several specific variables measured similar things with slight variation in the specific variable definition. Measures of accessibility or remoteness, including presence or distance to paved or any roads, as well as access to transportation or distance to a larger town, indicated that more remote areas or those with less access to roads had higher TF prevalence (Fig 2) (two of five associations were statistically significant) [30,51,60]. The variables for no medical care centre or pharmacy present in a village or increased distance from medical care were also associated with higher TF prevalence (two of six associations were statistically significant) [30,42,51,57]. On the other hand, the presence of a school, increased school density, smaller distance to school, or more school attendance in an area were associated with lower TF prevalence [57,60], though three of the four results came from the same study using different specific variables; all associations were statistically significant. Water or sanitation use, coverage, access, and availability were measured with nine different specific variables in nine studies. The overall trend indicates that less sanitation access (all associations were statistically significant) [38,53,60] and worse water access (two of three associations were statistically significant) [38,42,60,62] were associated with higher TF prevalence.

No infrastructure category variables were included in TT analyses.

### 3.3.5 Associated factor trends: Socioeconomic

The socioeconomic status category for studies with TF as main outcome included the fewest variables: six variables across two studies [57,60], and none met the threshold for inclusion in the synthesis of the results. See S2 Table for full results.

Smith *et al*. 2015 was the only study examining TT prevalence that included a socioeconomic associated factor [61].

### 3.4 Spatial analysis and trachoma studies

There were 14 studies identified in this systematic review that included spatial analysis [30,32–35,37,46,47,49,54,56,58,60,61] (Table 5). Of these, ten focused on TF only [30, 32–35,47,49,54,56,60], while two examined TT only [37,61], one looked at both TF and TT [58], and one included both a measure of TF and ocular Ct infection [46]. The level of analysis for the studies was predominately survey cluster (defined as village or community or school-based), while two studies performed household-level spatial analysis examining spatial trends within a survey cluster [32,54], and two included district-level analysis [30,33]. Most studies focused on a single country, while one TT study included ten countries [37]. As with the associated factor analysis, Ethiopia [30,33,37] and Brazil [47,49,56] were the most common countries included, with three studies each.

**Table 5:** Spatial analysis studies by trachoma outcome, analysis level, and analysis type.

References			Altherr, F. 2019 [30]	Bailey, R. 1989 [32]	Bero, B. 2016 [33]	Clements, A. 2010 [34]	Edwards, T. 2012 [35]	Flueckiger, R. 2019 [37]	Last, A. 2017 [46]	Luna, E. 1992 [47]	Macharelli, C. 2013 [49]	Polack, S. 2005 [54]	Schellini, A. 2010 [56]	Schemann, J. 2007 [58]	Smith, J. L. 2014 [60]	Smith, J. L. 2015 [61]
**Trachoma Outcome**		**TF**	X	X	X	X	X		X*	X	X	X	X	X	X	
		**TT**			X			X						X		X
**Analysis Level**		**District(s)**	X		X											
		**Cluster/School**	X			X	X	X	X	X	X		X	X	X	X
		**Households**		X								X				
**Analysis Type**	**Exploratory Spatial Data Analysis**	**Global Moran’s I**	X						X		X		X			
		**Local Moran’s I**			X				X		X		X		X	
		Getis-Ord Gi*	X													
		**Semivariogram**				X	X	X	X						X	
		**Clustering/Correlogram**		X								X				
		**Kulldorf spatial scan statistic**										X				
	**Interpolation**	**Kriging**				X										
		**Kernel estimate**									X					
		**Spatial interpolation (unspecified)**												X		
	**Modelling**	**Bayesian**													X	X
		**Generalized Linear Model**								X						
		**Mixed or random effect regression**					X		X			X				
		**Binomial logistic**						X								

TF: Trachomatous inflammation—follicular, TT: Trachomatous trichiasis, X*: TF & Ocular Chlamydia trachomatis infection

We grouped the spatial methods used in the studies into three main categories: exploratory spatial data analysis (ESDA), interpolation, and modelling. Half of studies (7 of 14) used spatial methods from at least two categories. The ESDA category was the most commonly used, employed in eleven studies [30,32–35,37,46,49,54,56,60], encompassing: Global Moran’s I, Local Moran’s I, Getis-Ord-Gi*, semivariogram, clustering analysis or correlograms, and Kulldorf spatial scan statistic (Table 5). The interpolation category includes three methods, each used once in three different studies [34,49,58]: kriging, kernel estimate, and unspecific spatial interpolation. Four analysis types were used across seven studies in the modelling category [35,37,46,47,54,60,61]; these included Bayesian, generalised linear model, mixed or random effect regression, and binomial logistic.

## 4 Discussion

This review identified factors associated with trachoma prevalence (measured by TF or TT or Ct infection) at community level in trachoma endemic countries within the published literature, as well as the methods used for spatial analysis of trachoma data in published manuscripts. The twenty-nine studies identified that explored trachoma-associated factors included in this systematic review showed the current interest and potential value of including these variables to enhance our understanding of trachoma epidemiology.

Key TF-associated factors that were included in multiple studies showed lower TF prevalence in areas with higher mean annual precipitation and higher mean annual temperatures. Areas at higher altitudes also had lower TF prevalence. Population and infrastructure variables were also present in multiple studies. Areas that were rural, were less accessible (further from a road or town), had fewer medical services, or had fewer schools, had a higher prevalence of TF. Higher TF prevalence was also associated with worse access to water and sanitation at the community level. As for TT, the bulk of the evidence comes from a single cross-country study by Flueckiger *et al*. [37], which examined TT-associated factors in ten countries. That study showed that areas with a high aridity index and decreased nightlight density had higher TT prevalence.

Our findings are consistent with the results of a previous review by Ramesh *et al*. [20], which focused solely on climatic and environmental variables; their results also indicated that higher TF prevalence was associated with lower mean annual precipitation and lower altitude. However, Ramesh *et al*. [20] found inconsistent results related to temperature, while the current review suggests that higher temperatures are associated with lower TF prevalence. Furthermore, other studies have shown that TF is associated with low access to water and sanitation often measured at the individual or household level [64,65]; this review suggests this finding to also be true at the community level.

Regarding the use of spatial methods in trachoma prevalence studies, 14 studies identified in this systematic review included spatial analysis. The ESDA methods were most common in the studies and were mainly used to indicate the spatial association or relationship between communities or districts in the included studies. Modelling methods were used in seven studies. Specifics varied for each, but general approaches were similar, including using spatially explicit covariates (associated factors) and including a variable that accounted for the spatial relationship in the data. Greater availability of standardised global or national demographic, socioeconomic, and infrastructural spatial covariates produced and published in recent years makes the inclusion of these important factors easier at small geographic scales, more standard across countries, and less reliant on specific countries sharing national statistical survey data. This is especially important since variables in the infrastructural and demographic category were underrepresented in this review but showed some trends toward being associated with trachoma.

Our systematic review has several potential limitations. First, as most of the 59 specific variables examined were only used in one or two studies in a single country, no definitive conclusions could be drawn. Furthermore, due to heterogeneity across studies, comparison across factors was also not possible. Second, predictive models as were used in the included studies have drawbacks including potential overfit, inappropriately narrow 95% CIs, and optimistically small p-values which can limit our ability to identify true associations. We dealt with this challenge by considering general trends as the main measure of association and potential statistical significance as a secondary input. Third, a focus on results published in peer-reviewed journals, such as the majority of data included herein, can allow publication bias to have an effect; analyses are more likely to be submitted for publication if they have more interesting results [66]: a label generally accorded to results reported as being statistically significant. For example, in a few manuscripts, one or more potentially associated factors were considered by researchers and mentioned as being used in the analysis in the methods section, but not included in the reported results. This may have been due to statistical insignificance or collinearity identified in the analysis process but was generally not explained in the publication. Furthermore, studies that take place in areas of higher prevalence or in areas that receive external donor support for trachoma elimination activities and have specific motivations for publication of study, may be more likely to be submitted for publication. Fourth, the results may not be generalisable as there is heterogeneity in results both within sub-Saharan Africa and between different regions. The studies which examined a single country included in this review were predominantly from sub-Saharan Africa (19 of 27), with nine of those studies in Ethiopia. The Americas and the Pacific Islands include areas endemic for trachoma with potentially different associated factors but which are relatively under-studied [67]. Furthermore, specific country contexts such as baseline trachoma prevalence or historical treatment coverage were not included in this review as potential moderating factors to observed associations. Fifth, there was diversity in study quality across included studies, limiting our ability to accurately compare results across different study types. This is especially true of data collected before 2012, when standard systems to increase quality control and quality assurance [68] in trachoma prevalence surveys had not yet been routinely applied.

Since 2012, countries working towards reaching the trachoma elimination goals have collected an enormous amount of standardised trachoma prevalence data with support from GTMP and its successor, Tropical Data. These data lend themselves well to further exploration of trachoma prevalence and its associated factors at local, national, and global scales, informing future programmatic needs and funding priorities. Even more detailed examination of associated factors using standardised data from multiple countries could provide an internally consistent examination of associations. Work using more multi-country data with a wide array of spatial covariates could determine how universal the conclusions from this review might be [37]. Another area where further work will be needed is in understanding the relationship between associated factors previously identified and ocular Ct infection or seropositivity. The latter measure of trachoma was only included in one of the spatial analysis studies included in this review, but is becoming more commonly measured in trachoma surveys [69].

The results of this systematic review can be used to aid our understanding of trachoma epidemiology, and to indicate areas of future research. The breadth and number of variables used across the 29 included studies indicate the interest of researchers in better understanding the community-level factors that are associated with trachoma prevalence. We have identified some key factors that are worth including as covariates in studies aimed at understanding the prevalence of trachoma. Researchers and programmatic decision-makers should consider the inclusion and potential influence of precipitation, temperature, and altitude along with variables related to ruralness, accessibility, access to medical services and schools, and community-level water and sanitation coverage. The use of spatial methods in trachoma studies is becoming increasingly common and is likely to yield progressively greater insights into the epidemiology and control of trachoma in the future.

## Supporting information

S1 Text Systematic Review Protocol. (PDF)

S2 Text PRISMA Checklist. (PDF)

S3 Text Database search terms. (PDF)

S4 Text Associated factor variables used in studies and data sources. (PDF)

S1 Table Specific variable results for TF and TT. (XLSX)

S2 Table Full results by study, country, analysis type, outcome, and specific variable. (XLSX)
