# Supplementary material for: Community-level trachoma ecological associations and the use of geospatial analysis methods: A systematic review
Source: PLoS Negl Trop Dis. 2022 Apr 8;16(4):e0010272. doi: 10.1371/journal.pntd.0010272 (PMC9020723; doi:10.1371/journal.pntd.0010272)
Supplement: S1 Text — (PDF) [file pntd.0010272.s001.pdf]

Trachoma community risk factors and spatial associations

Clara Burgert, Pia Mingkwan, Jeremiah Ngondi, Emma Harding-Esch

Citation

Clara Burgert, Pia Mingkwan, Jeremiah Ngondi, Emma Harding-Esch. Trachoma community risk factors and spatial associations. PROSPERO 2020 CRD42020191718 Available from:
https://www.crd.york.ac.uk/prospero/display_record.php?ID=CRD42020191718

Review question

Are environmental, climate, sociodemographic, socioeconomic, geographic, or spatial proximity factors associated with trachoma prevalence in communities where trachoma prevalence has been assessed within trachoma endemic countries?

Searches

Database:
Embase (Elsevier/Ovid)
Global Health (CABI, Ovid)

MEDLINE (US Medical Library/Ovid)

Dissertations & Theses Global

Web of Science (Clarivate Analytics)

Language: English, French, Spanish, or Portuguese

Publication period: January 1, 1950 to May 31, 2020

Please refer to protocol for search terms.

Types of study to be included

randomised control trials (RCTs), observational studies, longitudinal studies, cross-sectional studies

Condition or domain being studied

Trachoma prevalence measured by active trachoma (trachomatous inflammation—follicular, TF), trachomatous trichiasis (TT), or ocular Chlamydia trachomatis infection (measured by a nucleic acid amplification-based test)

Participants/population

Communities/villages/clusters/EUs where trachoma prevalence has been assessed using sample size and sampling approach adequate to provide a good prevalence estimate

Intervention(s), exposure(s)

Environmental, climate, sociodemographic, socioeconomic, geographic, or spatial proximity factors

Comparator(s)/control

Not applicable

Main outcome(s)

Identify key factors associated with trachoma in the literature that may be included as covariates in future geospatial analysis and their potential impact on trachoma outcomes

* Measures of effect

odds ratios

Additional outcome(s)

- Identify any geospatial raster surfaces previously used in trachoma studies (e.g. WorldPop Population Density Surface 2015, NASA MODIS NDVI)

-Identify geospatial methods used in previous trachoma studies (e.g. Moran's I, LISA, R PrevMap)

* Measures of effect

None

Data extraction (selection and coding)

Two-person review of title/abstracts/full text for inclusion (Clara Burgert and Research Assistant- Pia Mingkwan). Third person tie breaker at each stage (Jeremiah Ngondi). Burgert will prepare searches and compile in EndNote. After removal of duplicates then title/abstract review will be done by Burgert and Mingkwan. Using inclusion criteria:

Inclusion Criteria:

1. Papers from January 1, 1950 to May 31, 2020 (included in search terms)

2. Paper reported in in English, French, Spanish, or Portuguese

3. Paper reporting results for human conditions

4. Papers reporting primary data collection results or secondary data analysis

5. Paper reporting on conditions related to the eyes specifically active trachoma (trachomatous inflammation—follicular, TF), trachomatous trichiasis (TT), or ocular Chlamydia trachomatis

6. Paper reporting trachoma prevalence in communities/villages/clusters/EUs

7. Papers reporting on trachoma prevalence measured at communities/villages/clusters/EUs level and associated risk factors measured at same level

Each included final manuscript will be reviewed and key information will be extracted into an excel table with the following fields:

• First Author

• Year

• Journal

• Country

• Population (N)

• Type of Study (Ecological, cross-sectional, case-control, etc.)

• Data years

• Level (individual, household, community, district)

• Variable Category (Climate, Environment, Population, SES, WASH, Other)

• Analyzed Variable(s)

• OR

• CIs & p-values

• Univariate or multivariate model

• Outcome (TF1-9, TT, etc)

• Diagnostic Test used

• Analysis and Confounding Control

• Excluded Variable(s)

• Main Risks of Bias

• Main Limitations of Analysis

• Main Limitations of Description

• Full citation

Data extraction will be performed by Burgert and reviewed by Ngondi.

Risk of bias (quality) assessment

Study quality and risk of bias will be assessed using the GRADE guidelines. The main characteristics to be assessed are the study design, analysis methods, the reliability of grading trachoma signs or testing ocular secretions, and risk factors assessed. The assessment will be performed by Burgert and reviewed by Ngondi.

Strategy for data synthesis

The final inclusion and exclusion of studies identified will be reported in a PRISMA flow diagram. Extracted data will be tabulated including population, study design, variable category, and study results. The data likely will not be synthesized quantitatively in a meta-analysis due to variation in study designs. If possible, odds ratios and simple effect measures will be included for context along with any controlling factors from the analysis. A systematic narrative synthesis will bring together the information provided in the text and tables. Results from the risk of bias assessments will be presented in a matrix and will be used to support understand and interpretation of results.

Tables to be included are:

• Review robustness of search criteria and inclusion/exclusion criteria (Comparison of amount of agreement between the 2 reviewers at each stage)

• Review of Quality matrix of GRADE information

• Review of Bias in studies and across studies

• Summary of associated factors

• Summary of spatial methods and spatial datasets used if applicable

Analysis of subgroups or subsets

None planned

Contact details for further information

Clara Burgert
cburgert@rti.org

Organisational affiliation of the review

London School of Hygiene and Tropical Medicine

Review team members and their organisational affiliations

Ms Clara Burgert. London School of Hygiene and Tropical Medicine
Ms Pia Mingkwan. RTI International
Dr Jeremiah Ngondi. RTI International
Emma Harding-Esch. London School of Hygiene and Tropical Medicine

Collaborators

Assistant/Associate Professor Emma Harding-Esch. London School of Hygiene and Tropical Medicine

Type and method of review

Systematic review

Anticipated or actual start date

29 June 2020

Anticipated completion date

30 September 2020

Funding sources/sponsors

RTI International

Conflicts of interest

Language

English

Country

England, United States of America

Stage of review

Review Ongoing

Subject index terms status

Subject indexing assigned by CRD

Subject index terms

Gonorrhea; Humans; Infant, Newborn; Infant, Newborn, Diseases; Risk Factors; Trachoma

Date of registration in PROSPERO

07 July 2020

Date of first submission

16 June 2020

Stage of review at time of this submission

Stage	Started	Completed
Preliminary searches	Yes	Yes
Piloting of the study selection process	Yes	Yes
Formal screening of search results against eligibility criteria	Yes	No
Data extraction	No	No
Risk of bias (quality) assessment	No	No
Data analysis	No	No

Revision note

Updated team members

The record owner confirms that the information they have supplied for this submission is accurate and complete and they understand that deliberate provision of inaccurate information or omission of data may be construed as scientific misconduct.

The record owner confirms that they will update the status of the review when it is completed and will add publication details in due course.

Versions

07 July 2020
13 September 2020

PROSPERO
This information has been provided by the named contact for this review. CRD has accepted this information in good faith and registered the review in PROSPERO. The registrant confirms that the information supplied for this submission is accurate and complete. CRD bears no responsibility or liability for the content of this registration record, any associated files or external websites.
