# Supplementary material for: Community-level trachoma ecological associations and the use of geospatial analysis methods: A systematic review
Source: PLoS Negl Trop Dis. 2022 Apr 8;16(4):e0010272. doi: 10.1371/journal.pntd.0010272 (PMC9020723; doi:10.1371/journal.pntd.0010272)
Supplement: S3 Text — (PDF) [file pntd.0010272.s003.pdf]

S3 Text: Database search terms

Database	Search Terms
Medline	(exp Trachoma/ OR trachoma*.mp. OR exp Trichiasis/ OR trichias*.mp. OR exp Chlamydia trachomatis/ OR "ocular chlamydia".mp.) AND
	(exp Socioeconomic Factors/ OR Housing/ OR housing.mp. OR refugees/ or vulnerable populations/ or working poor/ OR exp Refugee Camps/ OR Crowding/ OR Crowd*.mp. OR Population Density/ OR Toilet Facilities/ OR latrine*.mp. OR toilet*.mp. OR Drinking Water/ OR Sanitation/ OR sanitation.mp. OR Poverty/ OR Poverty Areas/ OR "Transients and Migrants"/ OR Rural Health/ OR Rural Population/ OR nightlight*.mp. OR night light*.mp. OR Medically Underserved Area/ OR underserved population*.mp. OR under served population*.mp. OR under-served population*.mp. OR nomad*.mp. OR socioeconomic.mp. OR exp Altitude/ OR altitude.mp. OR elevation*.mp. OR exp Climate/ OR climat*.mp. OR exp Weather/ OR weather.mp. OR meteorol*.mp. OR Seasons/ OR season*.mp. OR humid*.mp. OR exp Temperature/ OR temperature*.mp. OR exp Climatic Processes/ OR rain*.mp. OR precipitation.mp. OR arid*.mp. OR desert.mp. OR semiarid*.mp. OR drought*.mp. OR dryland.mp. OR sahel*.mp. OR exp Soil/ OR soil*.mp. OR savanna*.mp. OR ecosystem/ or forests/ or grassland/ or wetlands/ OR ecolog*.mp. OR ecosystem*.mp. OR ecozone*.mp. OR flood*.mp. OR wind*.mp. OR heat*.mp. OR Livestock/ OR livestock.mp. OR typhoon*.mp. OR monsoon*.mp. OR Environmental Exposure/ OR Geography/ OR exp Geography, Medical/ OR models, spatial interaction/ OR exp spatial analysis/ OR Geographic Information Systems/ OR exp cluster analysis/ OR Remote Sensing Technology/ OR raster*.mp. OR hot spot*.mp. OR risk map*.mp. OR spatial covariate*.mp. OR spatial association*.mp. OR spatial distribution*.mp. OR getis ord.mp. OR moran*.mp.)
Embase	(exp trachoma/ OR trachoma*.mp. OR exp trichiasis/ OR trichias*.mp. OR exp Chlamydia trachomatis/ OR ocular chlamydia.mp.) AND
	(exp socioeconomics/ OR housing/ OR housing.mp. OR exp refugee/ OR exp vulnerable population/ OR exp wOR king poOR / OR exp refugee camp/ OR exp "crowding (area)"/ OR crowd*.mp. OR exp population density/ OR latrine*.mp. OR toilet*.mp. OR exp drinking water/ OR sanitation/ OR sanitation.mp. OR poverty/ OR migrant/ OR exp rural area/ OR rural health/ OR exp rural population/ OR nightlight*.mp. OR night light*.mp. OR medically underserved/ OR underserved population*.mp. OR under served population*.mp. OR under-served population*.mp. OR nomad*.mp. OR socioeconomic.mp. OR exp altitude/ OR altitude*.mp. OR elevation*.mp. OR exp climate/ OR climat*.mp. OR exp weather/ OR weather.mp. OR meteOR ol*.mp. OR exp season/ OR season*.mp. OR humid*.mp. OR exp temperature/ OR temperature*.mp. OR rain*.mp. OR precipitation.mp. OR arid*.mp. OR desert.mp. OR semiarid*.mp. OR drought*.mp. OR dryland.mp. OR sahel*.mp. OR exp soil/ OR savanna/ OR savanna*.mp. OR ecosystem/ OR exp land biome/ OR ecolog*.mp. OR ecosystem*.mp. OR ecozone*.mp. OR flood*.mp. OR wind*.mp. OR heat*.mp. OR livestock/ OR livestock.mp. OR typhoon*.mp. OR monsoon*.mp. OR environmental exposure/ OR geography/ OR exp medical geography/ OR exp spatial analysis/ OR spatial autocOR relation analysis/ OR geographic infOR mation system/ OR exp cluster analysis/ OR exp remote sensing/ OR raster*.mp. OR hot spot*.mp. OR risk map*.mp. OR spatial covariate*.mp. OR spatial association*.mp. OR spatial distribution*.mp. OR getis OR d.mp. OR moran*.mp.)

Database	Search Terms
Global Health	(exp trachoma/ OR trachoma*.mp. OR trichiasis.mp. OR exp Chlamydia trachomatis/ OR ocular chlamydia.mp.) AND
	(exp socioeconomic status/ OR housing/ OR housing.mp. OR exp refugees/ OR vulnerable population*.mp. OR working poor.mp. OR refugee camps.mp. OR exp crowding/ OR crowd*.mp. OR population density/ OR exp toilets/ OR exp latrines/ OR latrine*.mp. OR toilet*.mp. OR exp drinking water/ OR exp sanitation/ OR sanitation.mp. OR exp poverty/ OR poverty.mp. OR exp migrants/ OR exp rural health/ OR exp rural population/ OR nightlight*.mp. OR night light*.mp. OR underserved population*.mp. OR under served population*.mp. OR under-served population*.mp. OR nomad*.mp. OR socioeconomic.mp. OR exp altitude/ OR altitude.mp. OR elevation.mp. OR exp climate/ OR climat*.mp. OR exp weather/ OR weather.mp. OR meteorol*.mp. OR exp seasons/ OR season*.mp. OR exp tropics/ OR humid*.mp. OR exp temperature/ OR temperature*.mp. OR exp climate change/ OR exp precipitation/ OR rain*.mp. OR precipitation.mp. OR exp land types/ OR exp climatic zones/ OR exp "soil types (climatic)"/ OR arid*.mp. OR desert.mp. OR semiarid*.mp. OR drought*.mp. OR dryland.mp. OR sahel.mp. OR soil*.mp. OR exp vegetation types/ OR savanna*.mp. OR exp ecosystems/ OR exp ecology/ OR ecolog*.mp. OR ecosystem*.mp. OR ecozone*.mp. OR flood*.mp. OR wind*.mp. OR heat*.mp. OR exp domestic animals/ OR livestock.mp. OR typhoon*.mp. OR monsoon*.mp. OR exp exposure/ OR environment*.mp. OR exp geography/ OR exp geographical distribution/ OR exp spatial distribution/ OR exp spatial variation/ OR exp geographical information systems/ OR social behaviour/ or exp clustering/ OR spatial analysis.mp. OR cluster analysis.mp. OR exp remote sensing/ OR raster*.mp. OR hot spot*.mp. OR risk map*.mp. OR spatial covariate*.mp. OR spatial association*.mp. OR spatial distribution*.mp. OR getis ord.mp. OR moran*.mp.)
Thesis Global	noft((Trachoma*) OR (trichias*) OR ("ocular chlamydia")) AND
	noft((housing) OR (refugee*) OR ("vulnerable populations") OR ("working poor") OR (Crowd*) OR ("Population density") OR (latrine*) OR (toilet*) OR ("Drinking water") OR (sanitation) OR (Poverty) OR (migrants) OR (Transients) OR (Rural) OR (nightlight*) OR ("night light*") OR ("underserved population*") OR ("under served population*") OR ("under-served population*") OR (nomad*) OR (socioeconomic) OR (altitude) OR (elevation*) OR (climat*) OR (weather) OR (meteorol*) OR (season*) OR (humid*) OR (temperature*) OR (rain*) OR (precipitation) OR (arid*) OR (desert) OR (semiarid*) OR (drought*) OR (dryland) OR (sahel*) OR (soil*) OR (savanna*) OR (ecosystem) OR (ecolog*) OR (ecosystem*) OR (ecozone*) OR (flood*) OR (wind*) OR (heat*) OR (livestock) OR (typhoon*) OR (monsoon*) OR (Environment*) OR (Geograph*) OR ("spatial interaction") OR ("spatial analysis") OR ("cluster analysis") OR ("Remote sensing") OR (raster*) OR (("hot spot" OR "hot spots")) OR ("risk map*") OR ("spatial covariate*") OR ("spatial association*") OR (("spatial distribution" OR "spatial distributions")) OR ("getis ord") OR (moran*) OR ("spatial determinant*"))
Web of Science	(TS=(trachoma*) OR TS=(trichias*) OR ts=(chlamydia trachoma*) OR ts=(ocular chlamydia)) *AND*

**Database**	**Search Terms**
	(ts=(housing) OR ts=(refugee*) OR ts=(vulnerable populations) OR ts=(working poor) OR ts=(Crowd*) OR ts=(Population density) OR ts=(latrine*) OR ts=(toilet*) OR ts=(Drinking water) OR ts=(sanitation) OR ts=(Poverty) OR ts=(migrants) OR ts=(Transients) OR ts=(Rural) OR ts=(nightlight*) OR ts=(night light*) OR ts=(underserved population*) OR ts=(under served population*) OR ts=(under-served population*) OR ts=(nomad*) OR ts=(socioeconomic) OR ts=(altitude) OR ts=(elevation*) OR ts=(climat*) OR ts=(weather) OR ts=(meteorol*) OR ts=(season*) OR ts=(humid*) OR ts=(temperature*) OR ts=(rain*) OR ts=(pre cipitation) OR ts=(arid*) OR ts=(desert) OR ts=(semiarid*) OR ts=(drought*) OR ts =(dryland) OR ts=(sahel*) OR ts=(soil*) OR ts=(savanna*) OR ts=(ecosystem) OR t s=(ecolog*) OR ts=(ecosystem*) OR ts=(ecozone*) OR ts=(flood*) OR ts=(wind*) O R ts=(heat*) OR ts=(livestock) OR ts=(typhoon*) OR ts=(monsoon*) OR ts=(Enviro nment*) OR ts=(Geograph*) OR ts=(spatial interaction) OR ts=(spatial analysis) OR ts=(cluster analysis) OR ts=(Remote sensing) OR ts=(hot spot*) OR ts=(risk map*) OR ts=(spatial covariate*) OR ts=(spatial association*) OR ts=(spatial distribution*) OR ts=(getis ord) OR ts=(moran*))
