# Supplementary material for: Community-level trachoma ecological associations and the use of geospatial analysis methods: A systematic review
Source: PLoS Negl Trop Dis. 2022 Apr 8;16(4):e0010272. doi: 10.1371/journal.pntd.0010272 (PMC9020723; doi:10.1371/journal.pntd.0010272)
Supplement: S4 Text — (PDF) [file pntd.0010272.s004.pdf]

S4 Text: Associated factor variables used in studies and data sources

Variable Category	Variable Group	Specific Variable	Data Sources
Climate	Air pressure	Air pressure	National Climatic Data Center (NCDC)
Climate	Aridity Index	Aridity Index	Consortium for Spatial Information (CGIAR-CSI) Global-Aridity and Global-PET Database
Climate	Precipitation	Mean annual precipitation	WorldClim, National Climatic Data Center (NCDC), Weather Stations
Climate	Precipitation	Monthly rain days	FAOCLIM
Climate	Relative humidity	Relative humidity	Weather Stations
Climate	Sunshine fraction	Sunshine fraction monthly	FAOCLIM
Climate	Temperature	Average Land Surface Temperature	Moderate Resolution Imaging Spectroradiometer (MODIS) on NASA's Terra satellite
Climate	Temperature	Maximum monthly temperature	FAOCLIM
Climate	Temperature	Maximum temperature in the hottest month	WorldClim
Climate	Temperature	Mean annual temperature	WorldClim, FAOCLIM, National Climatic Data Center (NCDC)
Climate	Temperature	Mean daily temperature	Weather Stations
Climate	Temperature	Monthly average land surface temperature	Moderate Resolution Imaging Spectroradiometer (MODIS) on NASA's Terra satellite
Climate	Temperature	Temperature annual maximum	WorldClim, National Climatic Data Center (NCDC)
Climate	Temperature	Temperature annual minimum	National Climatic Data Center (NCDC)
Climate	Temperature	Variance Land surface temperature	Moderate Resolution Imaging Spectroradiometer (MODIS) on NASA's Terra satellite
Demographic	Population	Crowding	Demographic and Health Survey (DHS)
Demographic	Population	Nightlights	Operational Linescan System instrument onboard a satellite of the Defence Meteorological Satellite Program
Demographic	Population	Population density	SEDAC's Gridded Population of the World (GPWv3)
Demographic	Population	Population size of a village	survey
Demographic	Residence	Indigenous community	survey

Variable Category	Variable Group	Specific Variable	Data Sources
Demographic	Residence	Ruralness	Global Rural-Urban Mapping Project (GRUMP), survey
Environment	Altitude	Altitude	Shuttle Radar Topography Mission (SRTM), GPS, survey, National Climatic Data Center (NCDC), WorldClim
Environment	Enhanced Vegetation Index	Enhanced Vegetation Index	Moderate Resolution Imaging Spectroradiometer NASA's Terra satellite (MODIS)
Environment	Environment zone	Land cover: Forest, Grassland, Savannah, Barren, Plains/Delta, Tropical	International Geosphere-Biosphere Programme (IGBP), UN Land Cover Classification System (LCCS) using ENVISAT satellite mission's MERIS sensor, MODIS, Africa Soil Information Service (AfSIS) using (MODIS), survey determined
Environment	Latitude	Latitude	GPS
Environment	Longitude	Longitude	GPS
Environment	Sand/soil fraction	Sand/soil fraction	ISRIC-World Soil Information project included in the Harmonized Soil Map of the World
Environment	Waterbody	Distance to water bodies	FAO
Environment	Waterbody	Surface water distance	FAO
Infrastructure	Access	Distance to a primary road	Digital Chart of the World (DIVA-GIS)
Infrastructure	Access	Distance to road	Digital Chart of the World (DIVA-GIS)
Infrastructure	Access	Paved Road present	survey
Infrastructure	Access	Transportation	survey
Infrastructure	Access	Village distance to town	survey
Infrastructure	Medical services	Dispensary present	survey
Infrastructure	Medical services	Distance to a medical centre	survey
Infrastructure	Medical services	Distance to a primary health Centre	survey
Infrastructure	Medical services	Health Centre present	survey
Infrastructure	Medical services	Pharmacy present	survey
Infrastructure	School	Distance to school	government data
Infrastructure	School	School attendance	government data

Variable Category	Variable Group	Specific Variable	Data Sources
Infrastructure	School	School density	government data
Infrastructure	School	School present	survey
Infrastructure	WASH	Sanitation-Access to improved sanitation	Demographic and Health Survey (DHS)
Infrastructure	WASH	Sanitation-Community sanitation usage	survey
Infrastructure	WASH	Sanitation-Open defecation	Demographic and Health Survey (DHS)
Infrastructure	WASH	Sanitation-Sanitation coverage in the cluster	survey
Infrastructure	WASH	Water-Access to an improved water source	Demographic and Health Survey (DHS)
Infrastructure	WASH	Water-Constructed water source	survey
Infrastructure	WASH	Water-Houses with a water source in the ward	survey
Infrastructure	WASH	Water-Tube well in the ward	survey
Infrastructure	WASH	Water-Water coverage in a cluster	survey
Socioeconomic	Farming	Cattle density	FAO
Socioeconomic	Farming	Irrigation	World Resource Institute
Socioeconomic	Farming	Ruminant density	FAO
Socioeconomic	Inequality	Inequality	other survey
Socioeconomic	Inequality	Poverty incidence	other survey
Socioeconomic	Social	Village association present	survey
Socioeconomic	Social	Women's association	survey
